# Temporal Bone Langerhans Cell Histiocytosis: An Uncommon Bilateral Presentation

**DOI:** 10.7759/cureus.12732

**Published:** 2021-01-16

**Authors:** Nojood E Alhaidri, Bader Alim, Nouf R Alrushaid, Hanadi Fatani, Ameen S Binnasser

**Affiliations:** 1 College of Medicine, King Saud University, King Saud University Medical City, Riyadh, SAU; 2 Department of Otolaryngology-Head and Neck Surgery, King Saud University Medical City, Riyadh, SAU; 3 Department of Histopathology, King Fahad Medical City, Riyadh, SAU; 4 Department of Otolaryngology-Head and Neck Surgery, King Fahad Medical City, Riyadh, SAU

**Keywords:** langerhans’cell histiocytosis, bilateral, temporal bone, otic

## Abstract

Langerhans cell histiocytosis (LCH) is a rare condition that presents clinically in various ways. The cause and subsequent development of LCH are idiopathic and not fully understood. This disease is mainly seen in childhood. It is rare to have bilateral temporal bone LCH as the initial presentation. LCH can affect many organs. However, the bilateral involvement of the temporal bone is very uncommon. Therefore, we believe documenting cases of this presentation can lead to a better understanding of the epidemiology and prevalence of the disease, which can contribute to its management planning.

A one-year-old boy was referred to a tertiary otolaryngology clinic with bilateral postauricular swelling, hearing loss, but no tenderness or ear discharge. During the patient evaluation, a CT scan was requested to further investigate the bilateral swelling, which showed bilateral bony destructive lesions in the temporal bone area. Next, the patient was scheduled for a biopsy of this lesion under general anesthesia. A biopsy of the right mastoid confirmed the diagnosis of LCH. The patient was started on LCH IV protocol for multifocal bone lesions (MFB) with special site induction. A follow-up fluorodeoxyglucose positron emission tomography/CT (FDG PET/CT) was performed on the whole body with the impression of mild interval improvement of the temporal bones’ masses bilaterally with stable bilateral cervical lymphadenopathy.

LCH is a rare pathology that requires comprehensive effort from various medical and surgical teams to reach the right diagnoses and start the patient on the best available treatment plan.

## Introduction

Langerhans cell histiocytosis (LCH) is a rare condition, and it presents clinically in various ways. The cause and subsequent development of LCH are idiopathic and not fully understood [1]. It is suggested that this disease might be seriously affected by predisposing factors that are viral or genetic. However, there is no conclusive evidence for it [1,2].

The incidence of LCH is approximately 5.4 cases for every one million people annually [1]. This disease is predominantly seen in the pediatric population [3]. There are around five to six LCH cases for every one million children [4]. LCH is more common in males [1], with a male-to-female ratio of 2:1 [5]. This disease can present in different age groups, with the patient age ranging from a few months to 15 years [4]; it is especially prevalent among children aged between one and four years [6], with a mean age of three years at presentation [7].

It is found that 14-61% of pediatric patients with LCH show involvement of the temporal bone and the ear [3]. However, other studies have stated that temporal bone is involved in 19-25% of LCH patients [8]. Moreover, 30% of the cases present bilaterally [9], more commonly when the disease is systemic or multifocal [10]. It is quite rare to have a bilateral temporal bone LCH as an initial presentation [11]. To the best of our knowledge, only 32 such cases have been reported in the literature in English so far.

The presentation of LCH with ear involvement can manifest as any of the following conditions: a polyp or mass in the canal of the external ear, swelling in the postauricular region, chronic inflammation of the external ear or middle ear, conductive hearing loss, vertigo, and sometimes, paralysis of the facial nerve [12], or, least likely, sensorineural hearing loss [8]. Swelling of soft tissue, conductive hearing loss, and otorrhea are seen more often in lesions of the temporal bone. Furthermore, conductive hearing loss occurs due to the erosion of the ossicles, soft tissue infiltration of the middle ear, or obstruction of the external auditory canal [8]. Nonetheless, patients presenting with exclusive ear symptoms constitute 25% of the patient population [3]. Generally, the involvement of the temporal bone is associated with systemic disease; thus, surgical excision is the best choice for management, with the option of adding radiotherapy, if indicated [13].

## Case presentation

A one-year-old boy with no known medical illnesses had bilateral postauricular swelling for five days, which was noticed by his parents in early March 2018. They initially sought medical help at the local hospital. A CT image showed a destructive soft tissue lesion in both the mastoid area and the petrous part of the temporal bone, causing narrowing of the ear canal.

The case was referred to a tertiary care center. Upon presentation to a tertiary care ENT clinic, the child's history was taken, revealing a healthy, full-term child of normal vaginal delivery with no health-related issues, except for the postauricular swelling in the absence of any other ear or vestibular symptoms. Perinatal and family histories were unremarkable.

Upon examination, he was found to be an alert and active child with no facial dysmorphic features. There was a bilateral swelling in the postauricular area, which was more prominent on the right side, with no tenderness or discharge. The ear canal was almost blocked; however, no discharge or any other swelling was seen. The rest of the ENT examination, including cranial nerves and flexible scope, was unremarkable.

**Figure 1:**
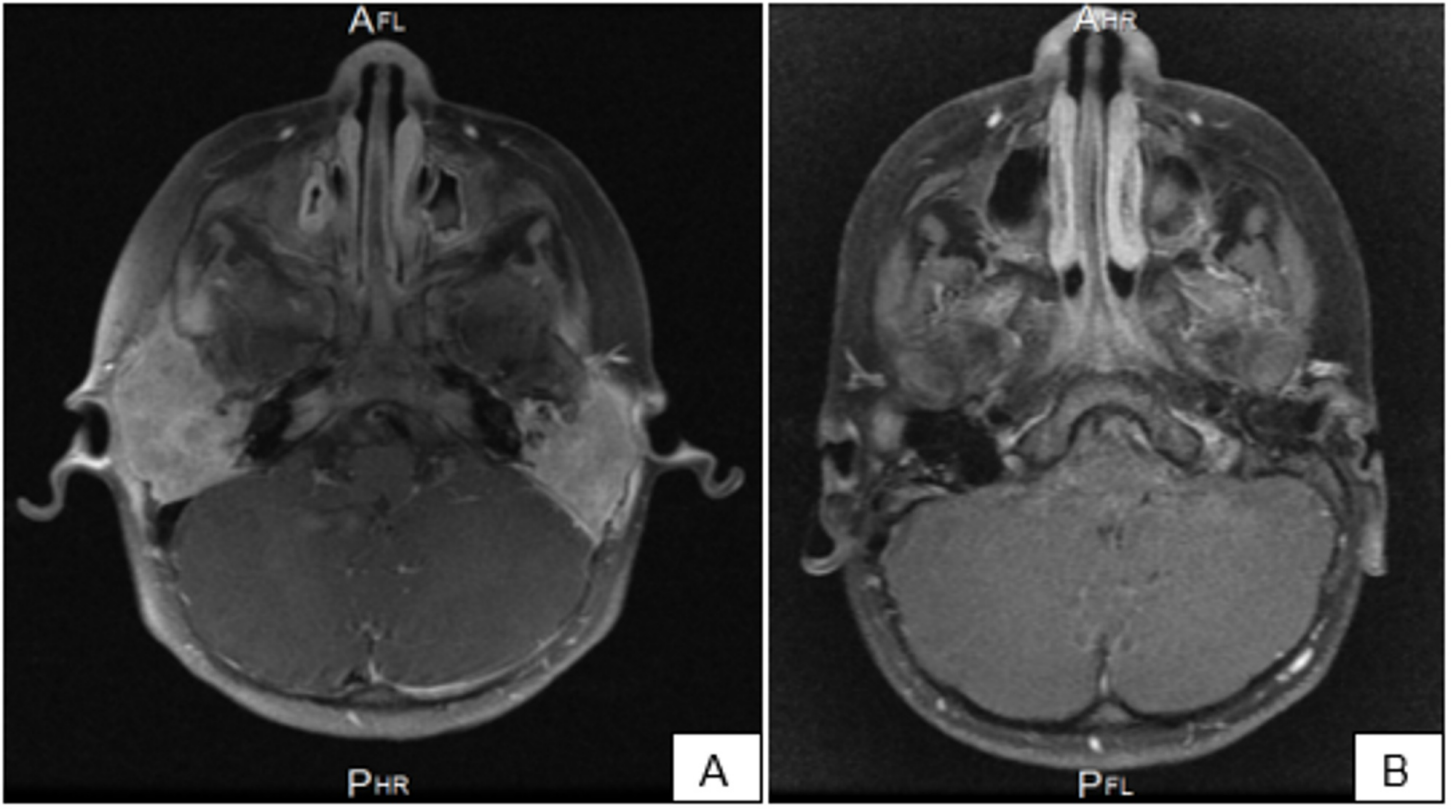
Axial view of post-contrasted T1 MRI images A: the image shows the heterogeneous enhancing lesion in the temporal bone area bilaterally; B: the image shows the decreased enhancement of the temporal bone lesions post-salvage protocol MRI: magnetic resonance imaging

The CT scan showed bilateral bony destructive lesions in the temporal bone area, which was larger on the right side, with a soft tissue component and intracranial extensions involving the external and internal ear cavities. An MRI of the temporal bone showed heterogeneous enhancement of the lesion bilaterally (Figure 1A). A positron emission tomography (PET) scan was performed to detect any other foci of the disease, and it showed a fluorodeoxyglucose (FDG)-avid bilateral temporal bone lesion with cervical lymphadenopathy and spleen involvement/reactive.

**Figure 2:**
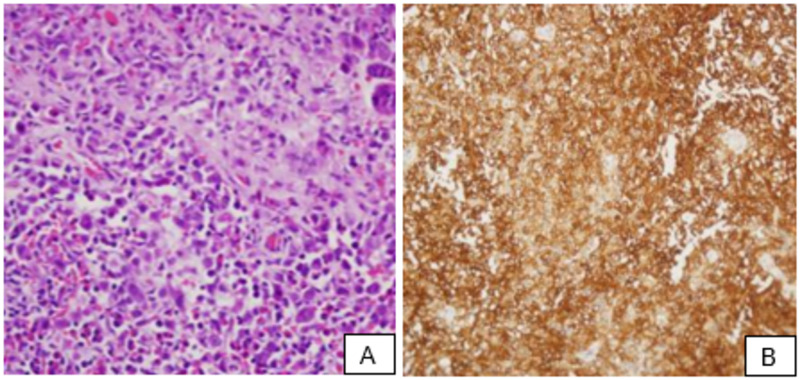
A: H&E shows Langerhans cell proliferation in dense eosinophilic infiltrate; B: immunohistochemistry with CD1a immunoreactive in tumor cells H&E: hematoxylin and eosin

After confirming the location and characteristics of the mass, the patient was taken for a mastoid lesion incisional biopsy under general anesthesia. A biopsy of the right mastoid showed the proliferation of Langerhans cells in a dense eosinophilic infiltrate (Figure 2A). Immunohistochemistry showed the following result: S100, CD68, and CD1a-positive (Figure 2B).

The patient’s case was discussed among the tumor board, and it was decided to start him on LCH IV protocol for multifocal bone lesions (MFB) with special site induction. In May 2018, the induction therapy was started for a duration of six weeks with vinblastine 6 mg/m^2^ IV bolus and prednisone 40 mg/m^2^/day orally as a weekly dose (reduction after the fourth week). Another six-week course of vinblastine and prednisone was given afterward. In June 2019, the last dose of maintenance therapy (vinblastine and prednisone every three weeks) was given, with a total duration of treatment of 52 weeks. Assessment with CT and PET scan was carried at the end of week 52 and showed active residual lesions in bilateral mastoid areas. Hence, in August 2019, a decision was made to start the salvage protocol with vincristine 1 mg/m^2^ IV over one minute once a week and cytarabine 500 mg/m^2^ twice daily for five days as a two-hour IV infusion. In January 2020, an assessment of CT of the chest and MRI brain and sinuses was performed, which showed further improvement of residual lesions and less enhancement (Figure 1B). Moreover, maintenance with 6-mercaptopurine (6-MP) 30 mg daily and methotrexate (MTX) 12.5 mg weekly was initiated.

A follow-up FDG PET/CT was performed for the whole body with the impression of mild interval improvement of the temporal bones masses bilaterally with stable bilateral cervical lymphadenopathy.

## Discussion

The present case of LCH presented with bilateral postauricular swelling, which was seen on imaging and confirmed by biopsy. The patient was managed by LCH IV protocol and followed up with a PET/CT scan, revealing a mild interval improvement bilaterally with stable cervical lymph nodes.

In a study by Coleman et al. [14], a two-year-old girl with no known medical illnesses had unilateral (left) postauricular swelling and erythema for 10 days with granulation-like changes in the posterior canal wall upon examination. A CT scan revealed bilateral temporal bone erosions in the petro-mastoid region, more prominent on the left side. The MRI also showed changes in the temporal bone bilaterally with soft tissue extension on the left. LCH was confirmed with a biopsy. Conductive hearing loss on the left side was demonstrated in audiometry. The management plan consisted of standard induction therapy, including vinblastine and prednisone, similar to our case scenario. On follow-up, a positive response to therapy was noted on imaging along with improvement in hearing, which was evident in audiometry.

Furthermore, another case of LCH was reported by Kleinjung et al. [11]. In this case, the child presented with a history of progressive hearing loss and imbalance for two months. A moderate vascular injection of the tympanic membranes was seen on otoscopy with no periauricular erythema or swelling. Audiometry revealed late responses from 80 to 100 dBHL, while the tympano-grams were flat on both sides. Auditory brainstem-evoked responses, paracentesis, and aspiration of fluid were performed under general anesthesia; no evidence for a retro cochlear cause of hearing loss was noted. The MRI showed symmetrical inflammatory changes and erosions of the temporal bone bilaterally, which are consistent with labyrinthitis. Destruction of the petrous bone and the labyrinthine bone was confirmed during exploratory surgery. The LCH diagnosis relied on immunohistochemistry results. Signs of further infiltration of the disease were negative on staging examinations. The case was further managed with systemic therapy, consisting of vinblastine and prednisone. On follow-up, a marked unilateral improvement was noted on the auditory brainstem-evoked responses.

Gupta et al. [8] have reported a unique case of successful cochlear implantation in a patient with bilateral temporal LCH. An eight-year-old girl presented with a six-month history of vertigo and progressive bilateral hearing loss for a year. Multiple lytic lesions in the temporal bones were seen on high-resolution CT. The diagnosis was confirmed with a biopsy. Persistent profound sensorineural hearing loss was evident bilaterally on pure tone audiometry. Similar to the cases mentioned above, this case was also managed with vinblastine and prednisone. Remission was determined after two years.

This disease is associated with an easily relapsing locally invasive growth pattern with the possibility of malignant tumor dissemination [1,11]. It is a condition of myeloid neoplasia that is inflammatory [15] and is distinguished by polyclonal abnormally proliferating Langerhans cells, resulting in the destruction and invasion of the tissue locally [8]. Furthermore, bone damage is the most common manifestation of this complicated disease. Temporal LCH might be confused with inflammatory lesions of the ear and malignant tumors due to its obscure characteristics [1]. Treatment options for this condition include surgical resection, radiotherapy, and steroid injections, while in unresponsive cases, aggressive chemotherapy is often introduced [13].

## Conclusions

LCH is a rare pathology that can affect many organs. A comprehensive effort involving various medical and surgical teams is warranted to reach the right diagnosis and start the patients on the best available treatment plan.
